# Use of concept mapping to characterize relationships among implementation strategies and assess their feasibility and importance: results from the Expert Recommendations for Implementing Change (ERIC) study

**DOI:** 10.1186/s13012-015-0295-0

**Published:** 2015-08-07

**Authors:** Thomas J. Waltz, Byron J. Powell, Monica M. Matthieu, Laura J. Damschroder, Matthew J. Chinman, Jeffrey L. Smith, Enola K. Proctor, JoAnn E. Kirchner

**Affiliations:** Department of Psychology, Eastern Michigan University, Ypsilanti, MI USA; Center for Clinical Management Research and Diabetes QUERI, VA Ann Arbor Healthcare System, Ann Arbor, MI USA; Department of Health Policy and Management, Gillings School of Global Public Health, University of North Carolina at Chapel Hill, Chapel Hill, NC USA; School of Social Work, College for Public Health & Social Justice, Saint Louis University, St. Louis, MO USA; Mental Health Quality Enhancement Research Initiative (QUERI), HSR&D, Department of Veterans Affairs, Little Rock, AR USA; VISN 4 MIRECC, VA Pittsburgh, Pittsburgh, PA USA; RAND Corporation, Pittsburgh, PA USA; Brown School, Washington University in St. Louis, St. Louis, MO USA; Department of Psychiatry, College of Medicine, University of Arkansas for Medical Sciences, Little Rock, AR USA; Central Arkansas Veterans Healthcare System, Department of Veterans Affairs, Little Rock, AR USA

**Keywords:** Concept mapping, Implementation research, Implementation strategies, Mental health, US Department of Veterans Affairs

## Abstract

**Background:**

Poor terminological consistency for core concepts in implementation science has been widely noted as an obstacle to effective meta-analyses. This inconsistency is also a barrier for those seeking guidance from the research literature when developing and planning implementation initiatives. The Expert Recommendations for Implementing Change (ERIC) study aims to address one area of terminological inconsistency: discrete implementation strategies involving one process or action used to support a practice change. The present report is on the second stage of the ERIC project that focuses on providing initial validation of the compilation of 73 implementation strategies that were identified in the first phase.

**Findings:**

Purposive sampling was used to recruit a panel of experts in implementation science and clinical practice (*N* = 35). These key stakeholders used concept mapping sorting and rating activities to place the 73 implementation strategies into similar groups and to rate each strategy’s relative importance and feasibility. Multidimensional scaling analysis provided a quantitative representation of the relationships among the strategies, all but one of which were found to be conceptually distinct from the others. Hierarchical cluster analysis supported organizing the 73 strategies into 9 categories. The ratings data reflect those strategies identified as the most important and feasible.

**Conclusions:**

This study provides initial validation of the implementation strategies within the ERIC compilation as being conceptually distinct. The categorization and strategy ratings of importance and feasibility may facilitate the search for, and selection of, strategies that are best suited for implementation efforts in a particular setting.

**Electronic supplementary material:**

The online version of this article (doi:10.1186/s13012-015-0295-0) contains supplementary material, which is available to authorized users.

## Background

Concerns about poor consistency in implementation science (IS) terminology have led researchers to characterize the field as a “Tower of Babel” [1]. Inconsistent terminology complicates literature searches, and researchers have found that search strategy yield and precision indices for implementation and quality improvement studies are moderate at best [2, 3]. This limits meta-analytic and replication efforts aimed at rigorously evaluating the effectiveness of implementation strategies and the value of existing literature for those enacting implementation initiatives. The science and practice of implementation would be greatly facilitated by a parsimonious nomenclature of conceptually distinct implementation strategies [2, 4–7].

Recently, Powell et al. [8] reviewed the health and mental health literature (including 41 compilations and reviews) and proposed a compilation of 68 discrete implementation strategies involving one action or process. This compilation served as the starting point for a subsequent multi-stage project called Expert Recommendations for Implementing Change (ERIC) [9]. The ERIC project’s first stage involved expert panelists (*N* = 71) using a modified Delphi process to revise the compilation, which resulted in an updated compilation of 73 discrete implementation strategies [10].

The aim of the ERIC project’s second stage, presented here, was to obtain preliminary validation of the compilation of 73 implementation strategies by studying the relationships between the strategies and obtaining relative importance and feasibility ratings for each strategy. The study of the relationships among the strategies supports the evaluation of whether the strategies are conceptually distinct from one another as well as how the strategies can be organized into conceptually relevant groupings. The former can also serve the practical purpose of making it easier for stakeholders to consider the range of implementation strategies by thematic cluster. The importance and feasibility ratings for the strategies provide insight into the perceived applicability of the strategies. It is of general interest which strategies have relatively high and low ratings by experts.

## Method

A purposive sampling procedure was used to recruit an expert panel of implementation science and clinical experts (*N* = 35) to participate in concept mapping and rating tasks [9, 10]. A detailed description of procedures has been published [9], and a summary is provided here. Concept mapping is a mixed-method procedure for engaging stakeholder groups in a structured conceptualization process [11]. This process supports visually representing the relationships among a set of related concepts and empirically clustering them into conceptually distinct categories and rating them on multiple dimensions.

The Concept Systems Global MAX™ [12] web platform was used for the panel’s sorting and rating tasks and data analysis. A more detailed introduction to concept mapping can be found in Trochim and Kane [13]. For the sorting task, participants were asked to sort virtual cards for each of the 73 strategies, accompanied by their definitions, into piles as they deemed appropriate. Participants were asked to rate each strategy for importance and feasibility ranging from 1 (relatively unimportant/not at all feasible) to 5 (extremely important/extremely feasible). These global ratings were prefaced by the following instructions: “Please select a number from 1 to 5 for each discrete implementation strategy to provide a rating in terms of how important (feasible) you think it is. Keep in mind that we are looking for *relative* importance (feasibility), use all the values in the rating scale to make distinctions.” Participants were able to select which set of activities they wanted to do first and were also able to work on the sorting and rating activities over multiple online sessions, at their convenience, before submitting their responses.

Multidimensional scaling and hierarchical cluster analyses were conducted to produce visual representations of the relationships among the strategies. Descriptive statistics for the importance and feasibility ratings were calculated. Each strategy’s importance and feasibility score was plotted on a graph. The resulting scatterplot was divided into four quadrants or “Go-zones” (e.g., I, II, III, IV) using the mean of each dimension. For example, quadrant I contains strategies that have values above the means for both dimensions. The Go-zone quadrants column in Table 1 reflects the combined relative importance and feasibility for each strategy.

**Table 1:** A summary of the 73 implementation strategies, organized by cluster with mean importance and feasibility ratings

		Importance	Feasibility	Go-zone quadrant
	Use evaluative and iterative strategies	4.19	4.01	–
4	Assess for readiness and identify barriers and facilitators	4.60	4.57	I
5	Audit and provide feedback	4.40	4.13	I
56	Purposefully reexamine the implementation	4.40	4.03	I
26	Develop and implement tools for quality monitoring	4.37	3.63	I
27	Develop and organize quality monitoring systems	4.33	3.37	I
23	Develop a formal implementation blueprint	4.30	4.47	I
18	Conduct local need assessment	4.27	4.33	I
61	Stage implementation scale up	3.97	3.77	I
46	Obtain and use patients/consumers and family feedback	3.67	3.80	I
14	Conduct cyclical small tests of change	3.63	4.03	I
	Provide interactive assistance	3.67	3.29	–
33	Facilitation	4.13	3.77	I
54	Provide local technical assistance	3.97	3.20	IV
53	Provide clinical supervision	3.83	3.10	IV
8	Centralize technical assistance	2.73	3.10	III
	Adapt and tailor to context	3.59	3.30	–
63	Tailor strategies	4.37	4.00	I
51	Promote adaptability	3.90	3.57	I
67	Use data experts	3.23	3.13	III
68	Use data warehousing techniques	2.87	2.50	III
	Develop stakeholder interrelationships	3.47	3.64	–
35	Identify and prepare champions	4.20	3.77	I
48	Organize clinician implementation team meetings	3.97	3.53	I
57	Recruit, designate, and train for leadership	3.93	3.20	IV
38	Inform local opinion leaders	3.90	4.03	I
6	Build a coalition	3.77	3.63	I
47	Obtain formal commitments	3.77	3.17	IV
36	Identify early adopters	3.70	3.70	I
17	Conduct local consensus discussions	3.63	4.07	I
7	Capture and share local knowledge	3.63	3.87	I
64	Use advisory boards and workgroups	3.40	3.87	I
65	Use an implementation advisor	3.30	3.70	I
45	Model and simulate change	3.30	3.20	II
72	Visit other sites	3.17	3.73	II
40	Involve executive boards	2.97	3.63	II
25	Develop an implementation glossary	2.87	4.57	II
24	Develop academic partnerships	2.83	3.40	II
52	Promote network weaving	2.70	2.77	III
	Train and educate stakeholders	3.43	3.93	–
19	Conduct ongoing training	4.17	3.87	I
55	Provide ongoing consultation	4.17	3.63	I
29	Develop educational materials	3.80	4.83	I
43	Make training dynamic	3.67	4.00	I
31	Distribute educational materials	3.50	4.77	I
71	Use train-the-trainer strategies	3.33	3.50	I
15	Conduct educational meetings	3.27	4.50	I
16	Conduct educational outreach visits	3.10	4.07	II
20	Create a learning collaborative	3.10	3.43	II
60	Shadow other experts	2.87	3.37	II
73	Work with educational institutions	2.73	3.30	II
	Support clinicians	3.23	3.06	–
32	Facilitate relay of clinical data to providers	4.17	3.43	I
58	Remind clinicians	3.23	3.77	II
30	Develop resource sharing agreements	3.07	3.13	III
59	Revise professional roles	3.00	2.30	III
21	Create new clinical teams	2.67	2.67	III
	Engage consumers	3.25	2.95	–
41	Involve patients/consumers and family members	3.87	3.63	I
39	Intervene with patients/consumers to enhance uptake and adherence	3.50	3.07	IV
50	Prepare patients/consumers to be active participants	3.40	3.03	IV
37	Increase demand	3.30	2.33	II
69	Use mass media	2.17	2.70	III
	Utilize financial strategies	2.86	2.09	–
34	Fund and contract for the clinical innovation	3.67	2.43	IV
1	Access new funding	3.57	2.40	IV
49	Place innovation on fee for service lists/formularies	3.40	2.10	IV
2	Alter incentive/allowance structures	3.17	2.23	III
42	Make billing easier	2.93	1.77	III
3	Alter patient/consumer fees	2.60	2.03	III
70	Use other payment schemes	2.30	1.87	III
28	Develop disincentives	2.17	2.13	III
66	Use capitated payments	1.97	1.80	III
	Change infrastructure	2.40	2.01	–
44	Mandate change	3.23	2.63	III
12	Change record systems	2.83	2.23	III
11	Change physical structure and equipment	2.60	2.27	III
22	Create or change credentialing and/or licensure standards	2.23	1.47	III
13	Change service sites	2.20	2.20	III
9	Change accreditation or membership requirements	2.17	1.80	III
62	Start a dissemination organization	2.03	2.13	III
10	Change liability laws	1.87	1.33	III

Strategies are organized by rank order of mean importance ratings from the highest to the lowest within each cluster. The importance rating scale ranged from 1 (relatively unimportant) to 5 (extremely important), and the feasibility scale ranged from 1 (not at all feasible) to 5 (extremely feasible). The rightmost column depicts the Go-zone quadrant into which each of the strategies falls based on the scale mean cutoffs (see Fig. 2). Go-zone quadrant I: Importance and feasibility are both above the scale means. Go-zone quadrant II: Importance rating is lower and feasibility rating is higher than the scale means. Go-zone quadrant III: Importance and feasibility ratings are both below scale means. Go-zone quadrant IV: Importance rating higher and feasibility lower than scale means

## Results

Experts who participated in the concept mapping and rating tasks and were affiliated with academic or healthcare institutions in the United States (*n* = 34) or in Canada (*n* = 1). Thirty-two of the 35 experts provided valid sorts (>75 % of strategies sorted), and 30 provided importance and feasibility ratings for all strategies. Sixty-three percent of participants had exclusive expertise in IS, 29 % were experts in both IS and clinical practice, and 8 % indicated clinical practice expertise only. Sixty-nine percent of participants had some affiliation with the US Department of Veterans Affairs (VA), most of whom also held academic appointments in social science or health-related schools or departments.

**Fig. 1:**
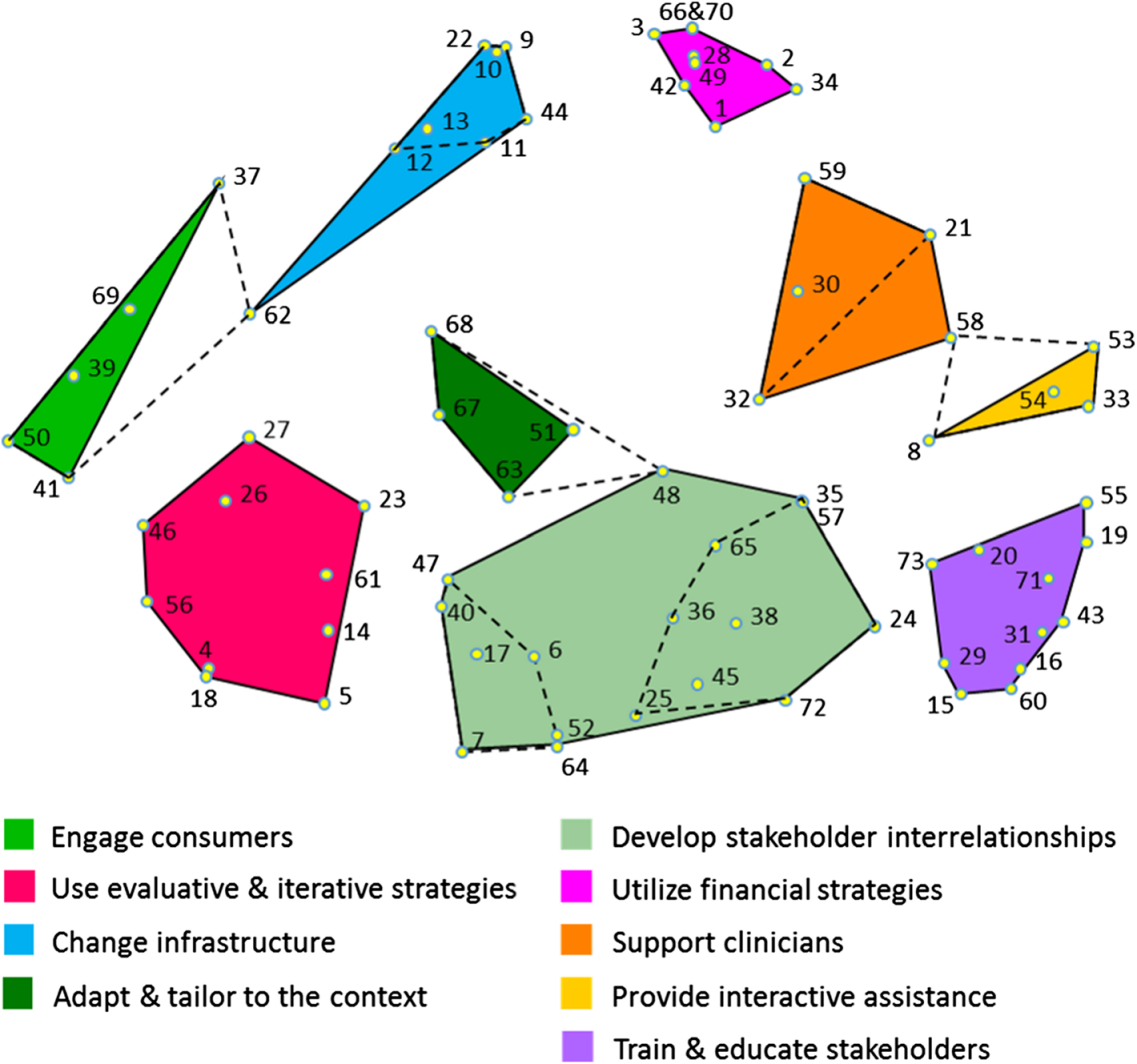
Point and cluster map of all 73 strategies identified in the ERIC process. The map reflects the product of an expert panel (valid response *n* = 32) sorting 73 discrete implementation strategies into groupings by similarity with each strategy being depicted by a *yellow dot* and accompanied by a *number* supporting cross-referencing to the strategies enumerated in Table 1. Spatial distances reflect how frequently the strategies were sorted together as similar. In general, the closer two points are together, the more frequently those strategies were sorted together. Strategies distal from one another were infrequently, if at all, sorted together. These spatial relationships are relative to the sorting data obtained in this study, and distances do not reflect an absolute relationship (i.e., a 5-mm distance in the present map does not reflect the same relationship as a 5-mm distance on a map from a different data set). The legend provides the label for each of the nine clusters of strategies. *Dotted lines* within the *Develop stakeholder interrelationships* cluster indicate how two separate clusters were merged into one large cluster due to conceptual similarity among their items. *Dotted lines* extending between other clusters archive the reassignment of strategies from their original cluster to a neighboring cluster to which there was a better conceptual fit (i.e., strategies #48, #58, and #62)

Figure 1 presents a point map that visually represents the relationships among the 73 implementation strategies, with each point on the map representing a strategy. The strategies are numbered to aid in cross-referencing the spatial relationships of the points on the map with their labels enumerated in Table 1. All but two strategies were sorted as being conceptually distinct. Strategies #66 (*Use capitated payments*) and #70 (*Use other payment schemes*) were always sorted together. Two other strategies were proximal to one another though they were sorted together by only 4 of 32 panelists (#35 *Identify and prepare champions* and #57 *Recruit, designate, and train for leadership*), indicating that they are more similar in how they relate with other strategies on the map, than they are directly similar to one another.

The final clusters were developed over 3 weeks of deliberations by the ERIC investigative team. A 13 cluster starting point was selected because it is one standard deviation above the mean number of clusters typically obtained in concept mapping [14]. In this study, 69 % of respondents sorted statements into 13 or fewer piles. We sequentially reviewed cluster merges and achieved consensus to merge clusters down to nine conceptually distinct clusters. For example, two clusters shown in pale green at the center bottom in Fig. 1 (separated by dashed lines) were merged to form a single cluster labeled *Develop stakeholder interrelationships*, as the original clusters were judged as not sufficiently conceptually distinct.

When the team reviewed the clusters for conceptual clarity, three proposals came forward to move individual strategies to neighboring clusters. First, #62 (*Start a dissemination organization*) was moved from the *Engage consumers* cluster to the *Change infrastructure* cluster, as it was judged more similar to infrastructure support for a practice change than engaging consumers. Second, #48 (*Organize clinician implementation team meetings*) was moved to the *Develop stakeholder interrelationships* cluster from *Adapt and tailor to the context*, as the former has greater interpersonal focus than the latter. And finally, #58 (*Remind clinicians*) was moved to the *Support clinicians* cluster from *Provide interactive assistance* because it is more administrative than interactive in focus. Unanimous consensus was reached for the final cluster arrangements. Additional file 1 provides a cluster-by-cluster visual tour of the concept map.

A multi-step process was used to determine labels for the final clusters. The list began with labels provided by expert panel members for their clusters that were most similar to the final cluster solutions. This list was supplemented with highly descriptive labels identified from the investigative team’s meeting minutes from cluster solution deliberations. Proposed criteria for developing cluster labels (Table 2) were introduced for team comment by one of the authors (LJD) along with suggested label revisions. These criteria were helpful in structuring iterative discussion among team members, the result of which was voted upon by the team and unanimously adopted.

**Table 2:** Guidelines for cluster labels

1	Short and elegant; simpler is better.
2	Easier for users to remember.
3	No redundancies (e.g., labeling with “…the implementation process” which is redundant in mentioning implementation because all these are for implementation; and redundant also because implementation is a process).
4	Not too short; enough description to evoke the general purpose/intent/theme underlying the cluster of techniques that are included.
5	Short enough to make it clear to users that they must look at the individual techniques within the cluster/package to know/understand the activities. A fully descriptive title may lead users to believe the label says it all.
6	Begin with a verb.
7	Command structure (definition: A type of sentence that gives advice or instructions or that expresses a request or command.). Not that these are requests/commands but they are certainly words of action-oriented advice.
8	Use layperson terms to the extent possible.

**Fig. 2:**
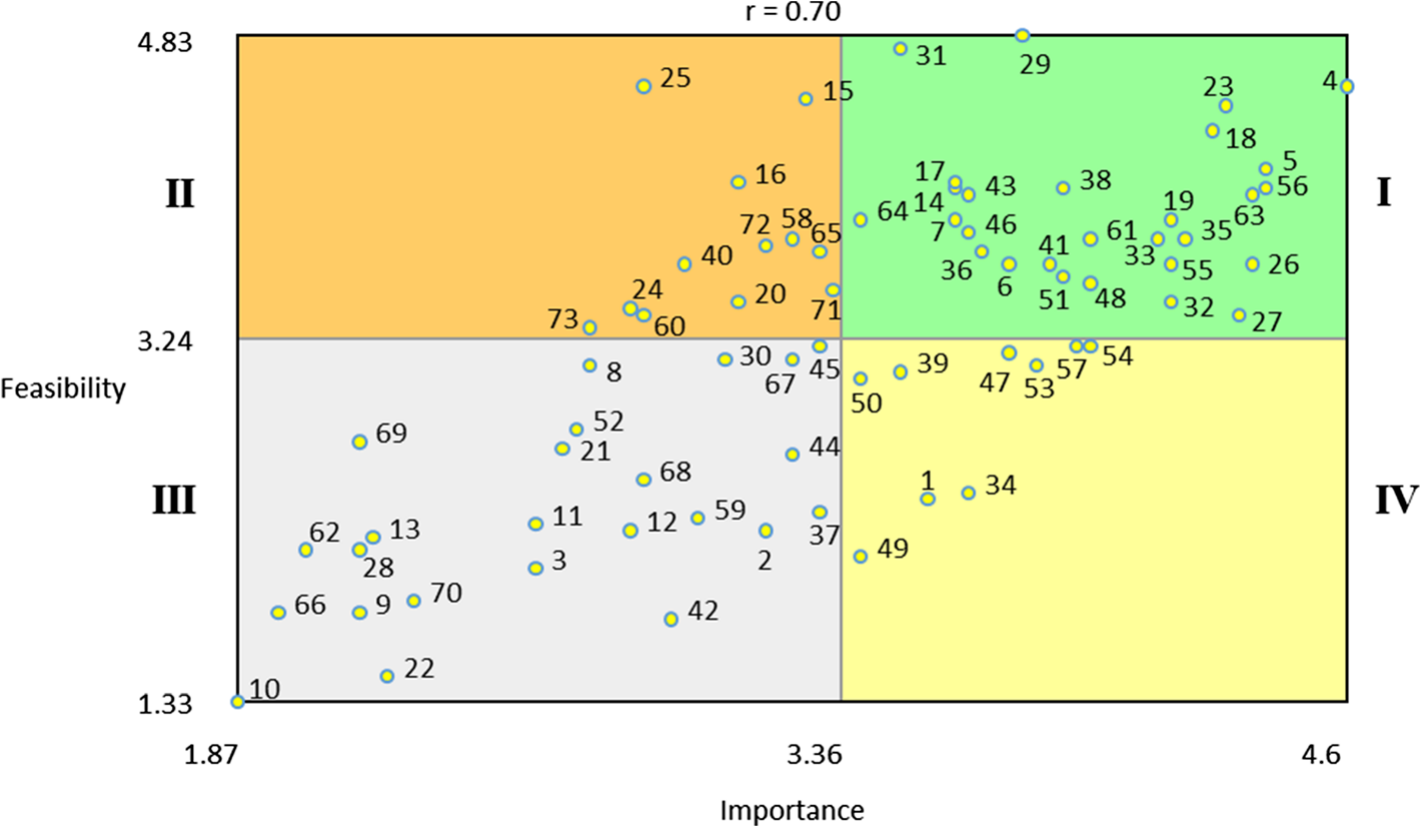
Go-zone plot for all 73 strategies based on expert ratings. *Note*. The range of the *x* and *y* axes reflect the mean values obtained for all 73 of the discrete implementation strategies for each of the rating scales. The plot is divided into quadrants on the basis of the overall mean values for each of the rating scales. Quadrant labels are depicted with *roman numerals* next to the plot. Strategies in *quadrant I* fall above the mean for both the importance and the feasibility ratings. Thus, these strategies are those where there was the highest consensus regarding their relative high importance and feasibility. Conversely, *quadrant III* reflects the strategies where there was consensus regarding their relative low importance and feasibility. *Quadrants II* and *IV* reflect strategies that were relatively high in feasibility or importance, respectively, but low on the other rating scale

Table 1 presents a summary of the 73 implementation strategies, organized by cluster with mean importance and feasibility ratings. There was a strong relationship (*r* = 0.7) between the feasibility and importance ratings, meaning that most strategies fell within either quadrant I (high importance and feasibility) or III (low importance/feasibility). However, there were still a number of strategies that were viewed as important but not as feasible (12 %, e.g., *Access new funding*), or feasible but less important (15 %, e.g., *Remind clinicians*). Clusters of strategies that are more immediate and concrete and are potentially more in the control of those tasked with supporting change (e.g., *Use evaluative and iterative strategies*, *Train and educate stakeholders*) tended to have higher importance and feasibility ratings. Clusters that are more strategic, but also potentially involve changing well-established systems (e.g., *Change infrastructure*, *Utilize financial strategies*), tended to have lower ratings. Figure 2 presents a graphic of the Go-zone data.

## Discussion

Results from this study provide initial validation for viewing the 73 implementation strategies as conceptually distinct. Cluster analyses of the concept mapping data support grouping strategies into nine clusters which have practical heuristic value for those looking to the ERIC compilation of implementation strategies for guidance. The importance and feasibility ratings for the strategies supported the formation of Go-zone quadrants that can be used to help decision makers prioritize which strategies to use when planning an implementation initiative.

While the concept mapping strategy used in this study represents a strong methodological approach to evaluating whether the 73 implementation strategies are conceptually distinct and organizing them by theme and potential applicability (i.e., Go-zone analysis), there are notable limitations. Recruitment had been restricted to the time zones within the continental United States to minimize scheduling conflicts for elements of the ERIC project that required real time interactions among participants. Thus, all but one of the 35 participants were from the United States, and 69 % had some affiliation with the VA. While concept maps with 30 or more participants are considered to be highly reliable [14], if stakeholders from outside the United States had practice contexts that alter the perceptions of these strategies interrelationships, or the ratings of their perceived importance and feasibility, different results may be obtained.

## Additional file

Additional file 1:
**A cluster-by-cluster visual tour of the concept map.**

## Abbreviations

ERIC: Expert Recommendations for Implementing Change
VA: US Department of Veterans Affairs
