# Supplementary material for: Use of concept mapping to characterize relationships among implementation strategies and assess their feasibility and importance: results from the Expert Recommendations for Implementing Change (ERIC) study
Source: Implement Sci. 2015 Aug 7;10:109. doi: 10.1186/s13012-015-0295-0 (PMC4527340; doi:10.1186/s13012-015-0295-0)
Supplement: Additional file 1: — A cluster-by-cluster visual tour of the concept map. [file 13012_2015_295_MOESM1_ESM.pdf]

Point Map

Strategies commonly sorted together as similar are physically closer to one another in the graphic below.

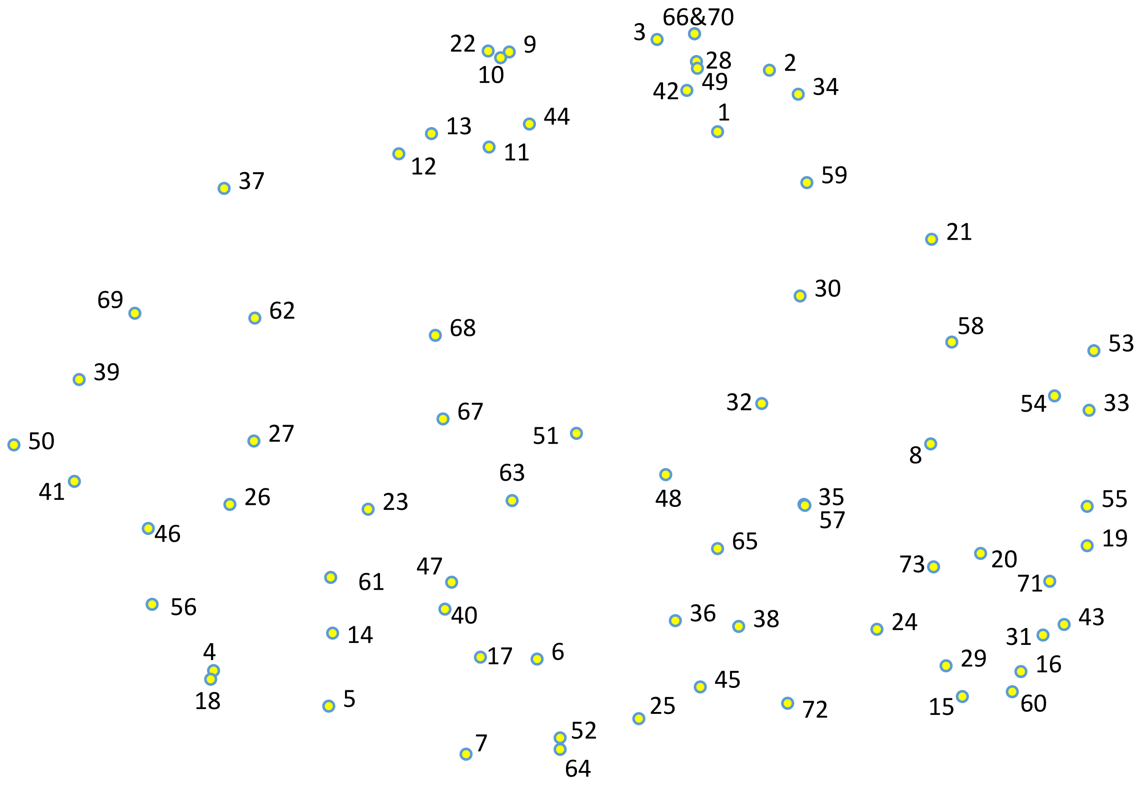

Final Solution

Strategies were clustered together by physical proximity into categories. Each cluster will be discussed in subsequent pages

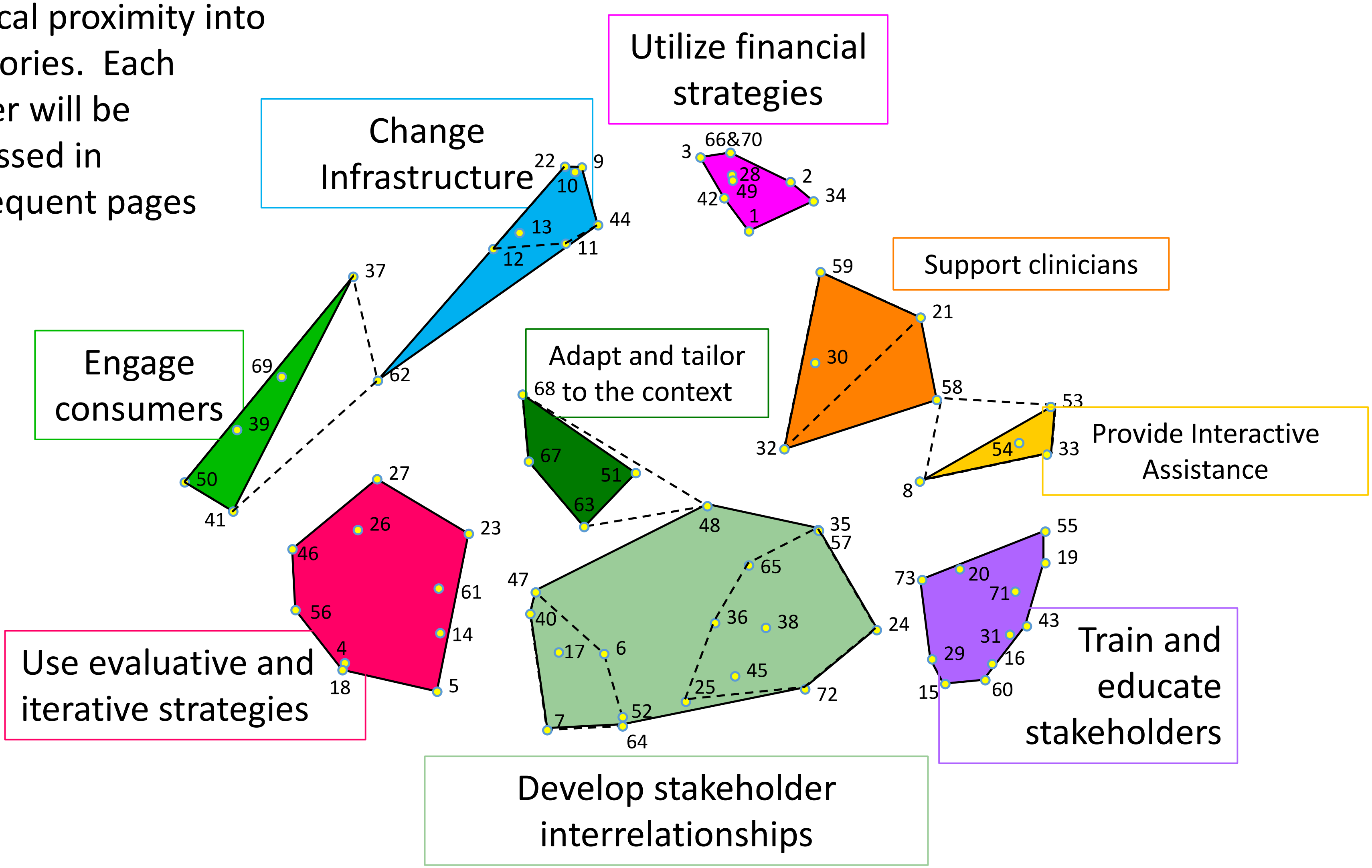

Use Evaluative and Iterative Strategies

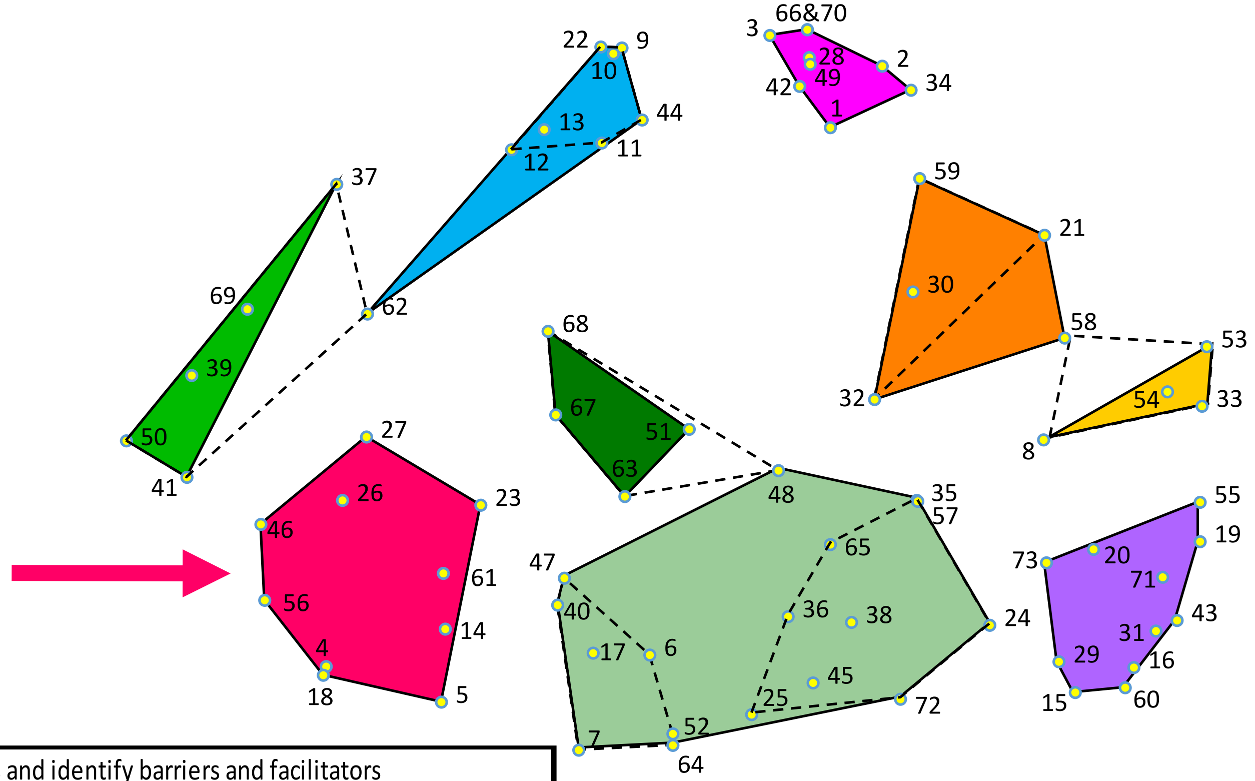

4	Assess for readiness and identify barriers and facilitators
5	Audit and provide feedback
14	Conduct cyclical small tests of change
18	Conduct local needs assessment
23	Develop a formal implementation blueprint
61	Stage implementation scale up

26	Develop and implement tools for quality monitoring
27	Develop and organize quality monitoring systems
46	Obtain and use patients/consumers and family feedback
56	Purposefully reexamine the implementation

Provide Interactive Assistance

8	Centralize technical assistance
33	Facilitation
53	Provide clinical supervision
54	Provide local technical assistance

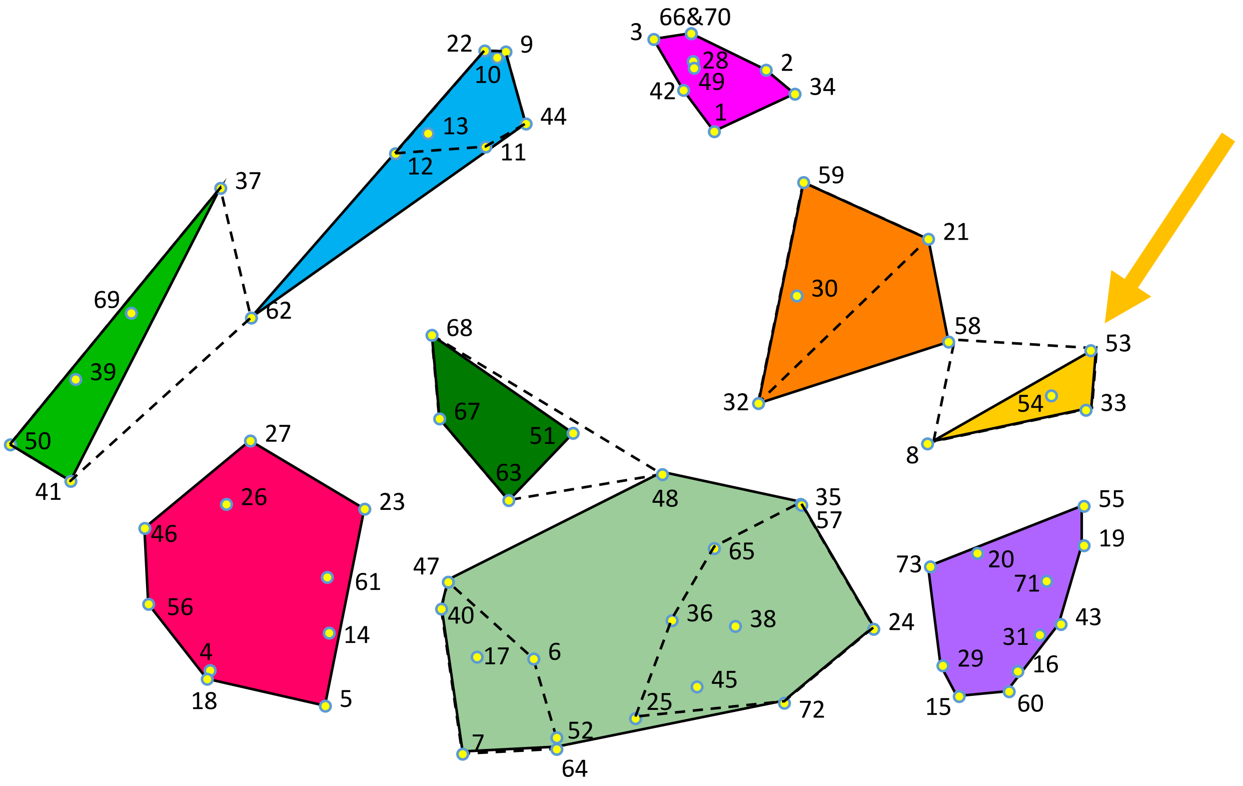

Adapt and Tailor to the Context

51	Promote adaptability
63	Tailor strategies
67	Use data experts
68	Use data warehousing techniques

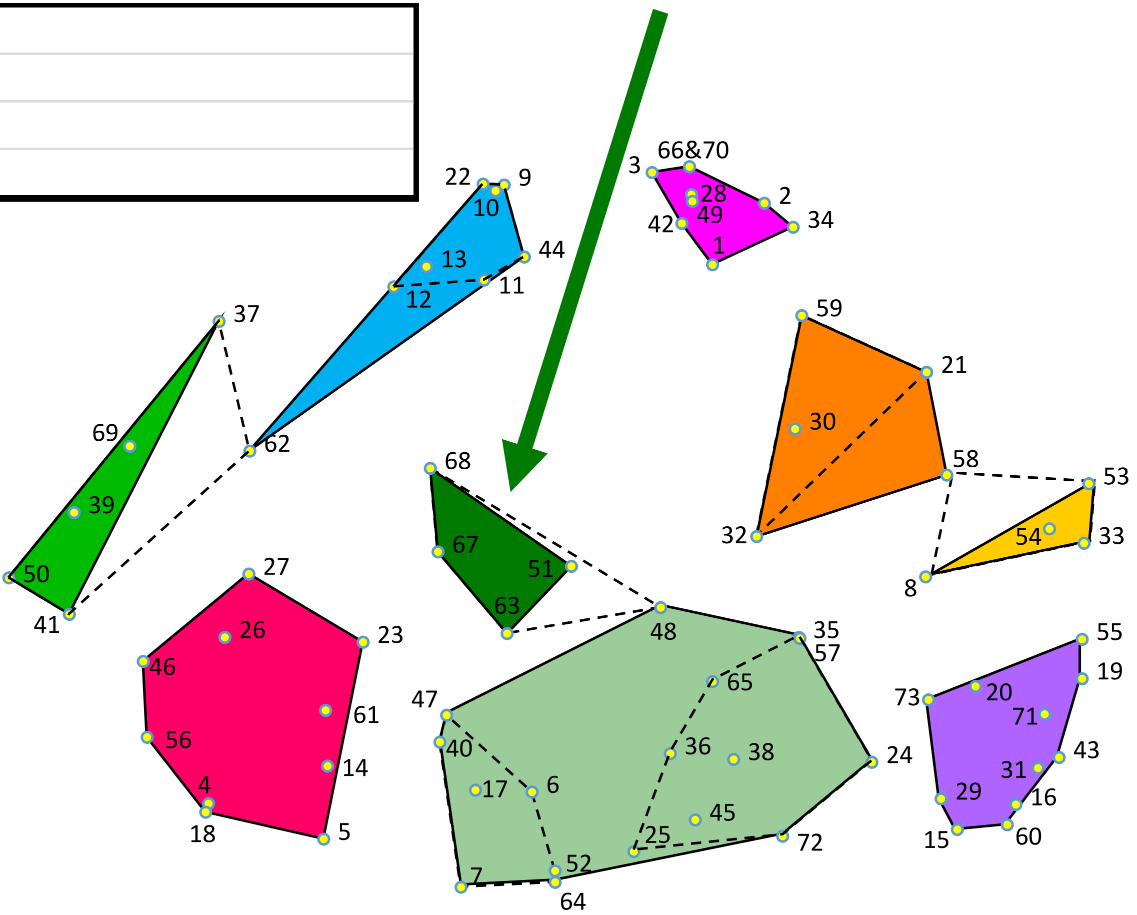

Develop Stakeholder Interrelationships

6	Build a coalition
7	Capture and share local knowledge
17	Conduct local consensus discussions
40	Involve executive boards
47	Obtain formal commitments
52	Promote network weaving
64	Use advisory boards and workgroups

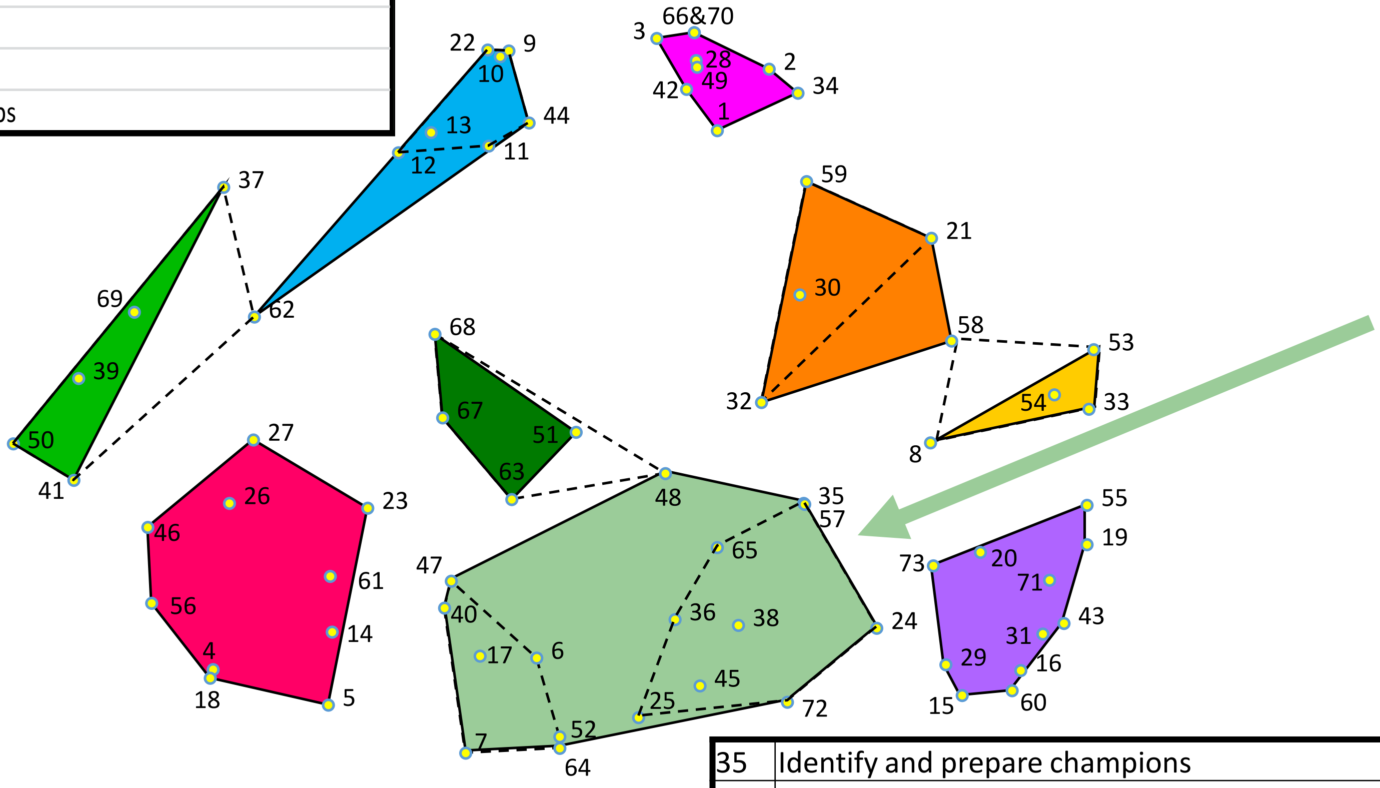

24	Develop academic partnerships
25	Develop an implementation glossary
36	Identify early adopters
38	Inform local opinion leaders

35	Identify and prepare champions
45	Model and simulate change
48	Organize clinician implementation team meetings
57	Recruit, designate, and train for leadership
65	Use an implementation advisor
72	Visit other sites

Train and Educate Stakeholders

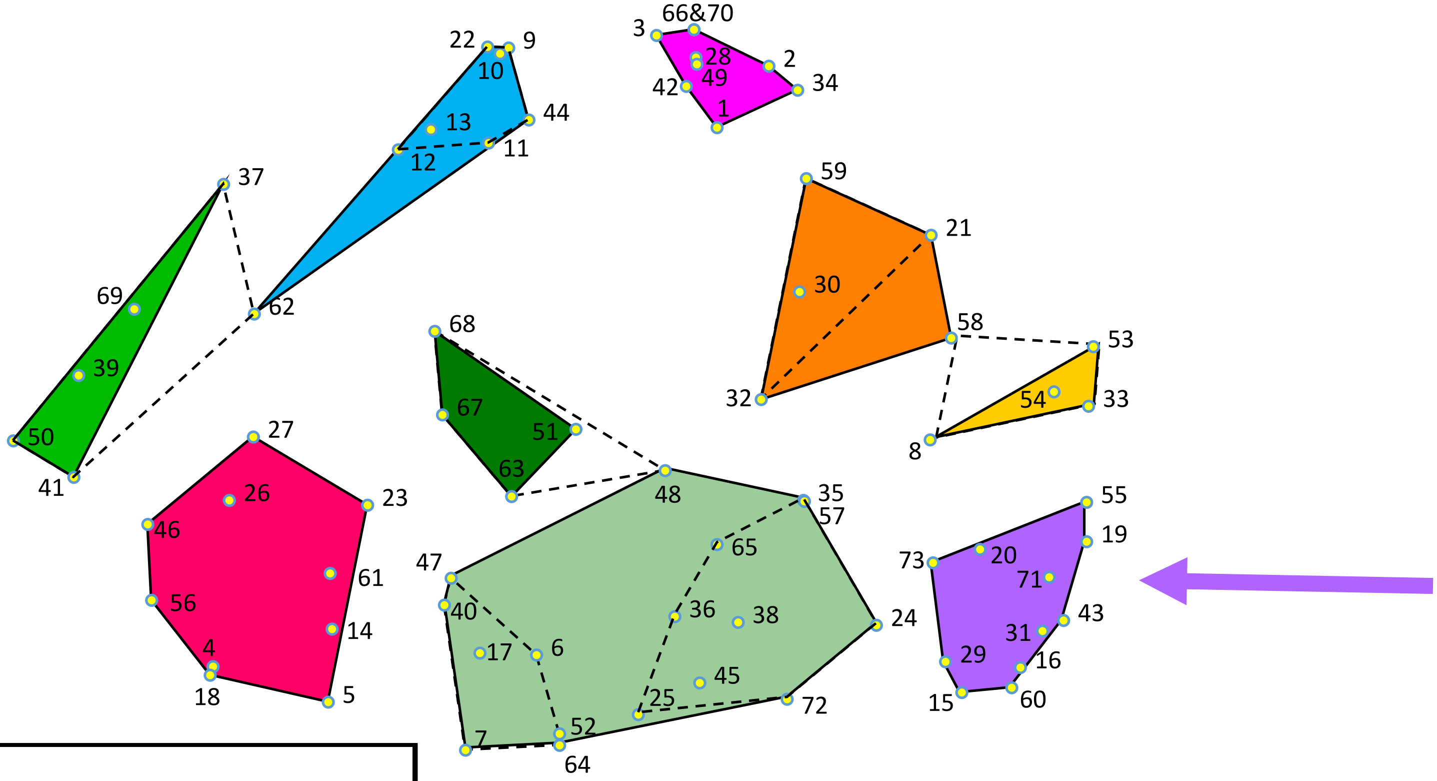

15	Conduct educational meetings
16	Conduct educational outreach visits
29	Develop educational materials
60	Shadow other experts
19	Conduct ongoing training
20	Create a learning collaborative

31	Distribute educational materials
43	Make training dynamic
55	Provide ongoing consultation
71	Use train-the-trainer strategies
73	Work with educational institutions

Support Clinicians

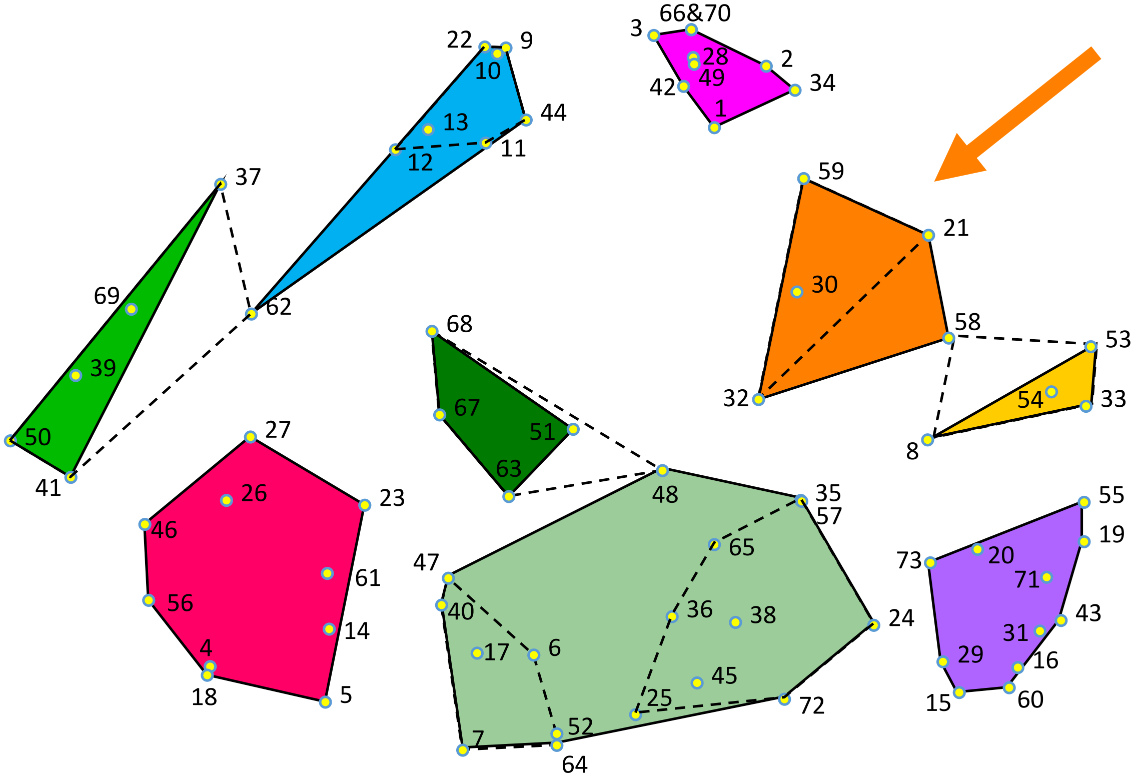

21	Create new clinical teams
30	Develop resource sharing agreements
32	Facilitate relay of clinical data to providers
58	Remind clinicians
59	Revise professional roles

Engage Consumers

37	Increase demand
39	Intervene with patients/consumers to enhance uptake and adherence
41	Involve patients/consumers and family members
50	Prepare patients/consumers to be active participants
69	Use mass media

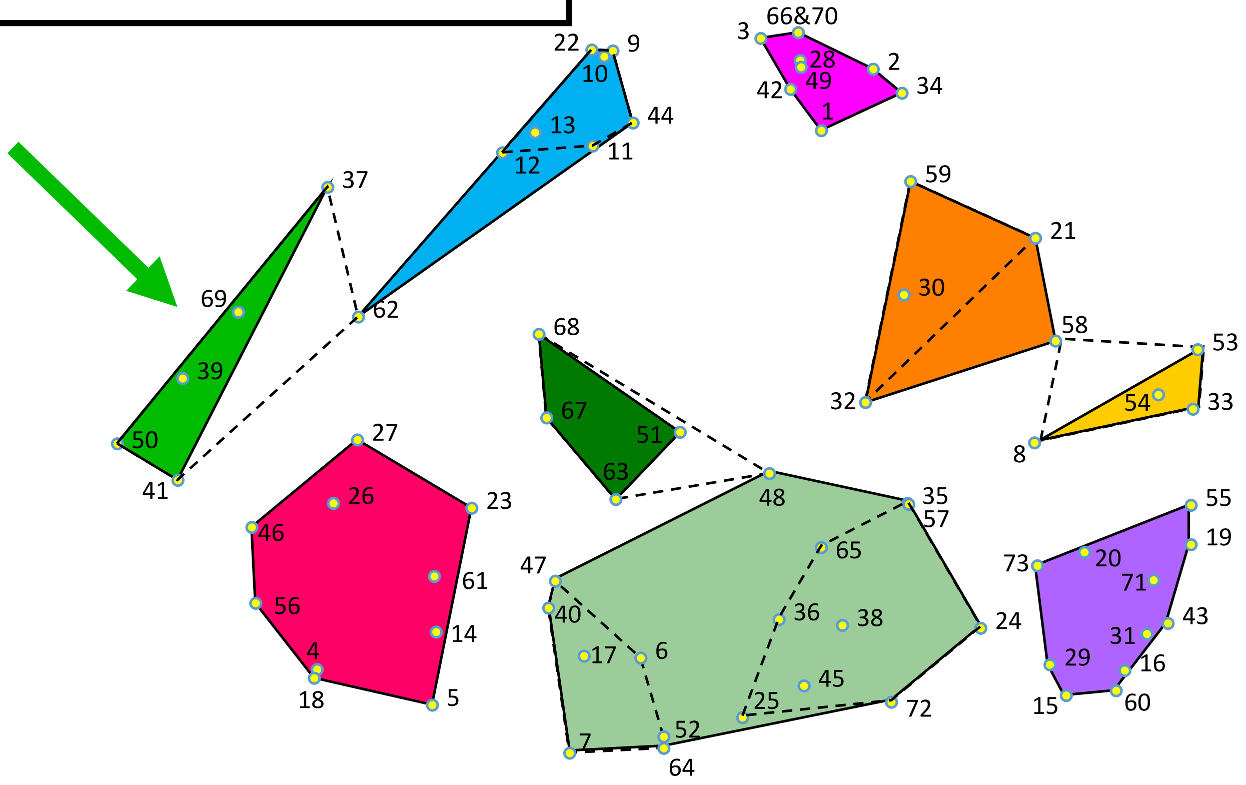

Utilize Financial Strategies

1	Access new funding
2	Alter incentive/allowance structures
3	Alter patient/consumer fees
28	Develop disincentives
34	Fund and contract for the clinical innovation

42	Make billing easier
49	Place innovation on fee for service lists/formularies
66	Use capitated payments
70	Use other payment schemes

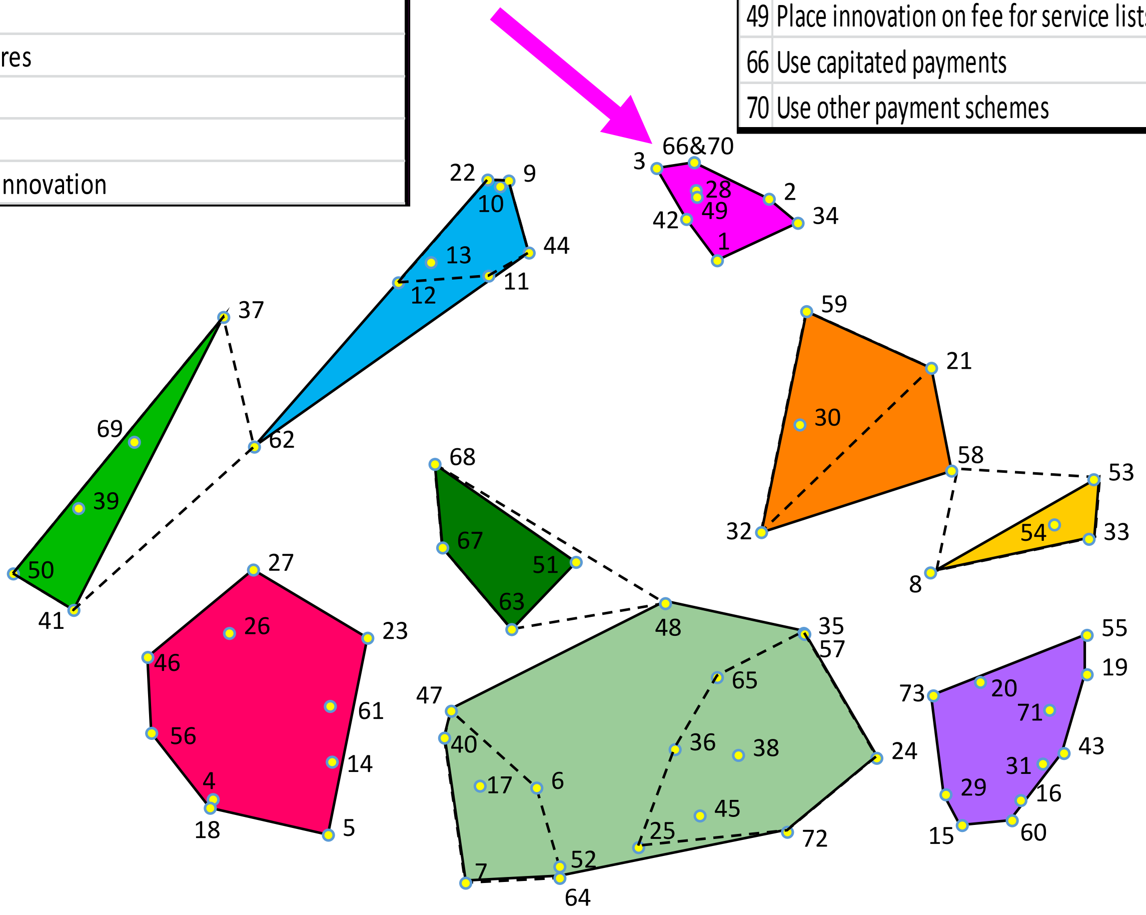

Change Infrastructure

9	Change accreditation or membership requirements
10	Change liability laws
11	Change physical structure and equipment
12	Change record systems
13	Change service sites
22	Create or change credentialing and/or licensure standards
44	Mandate change
62	Start a dissemination organization

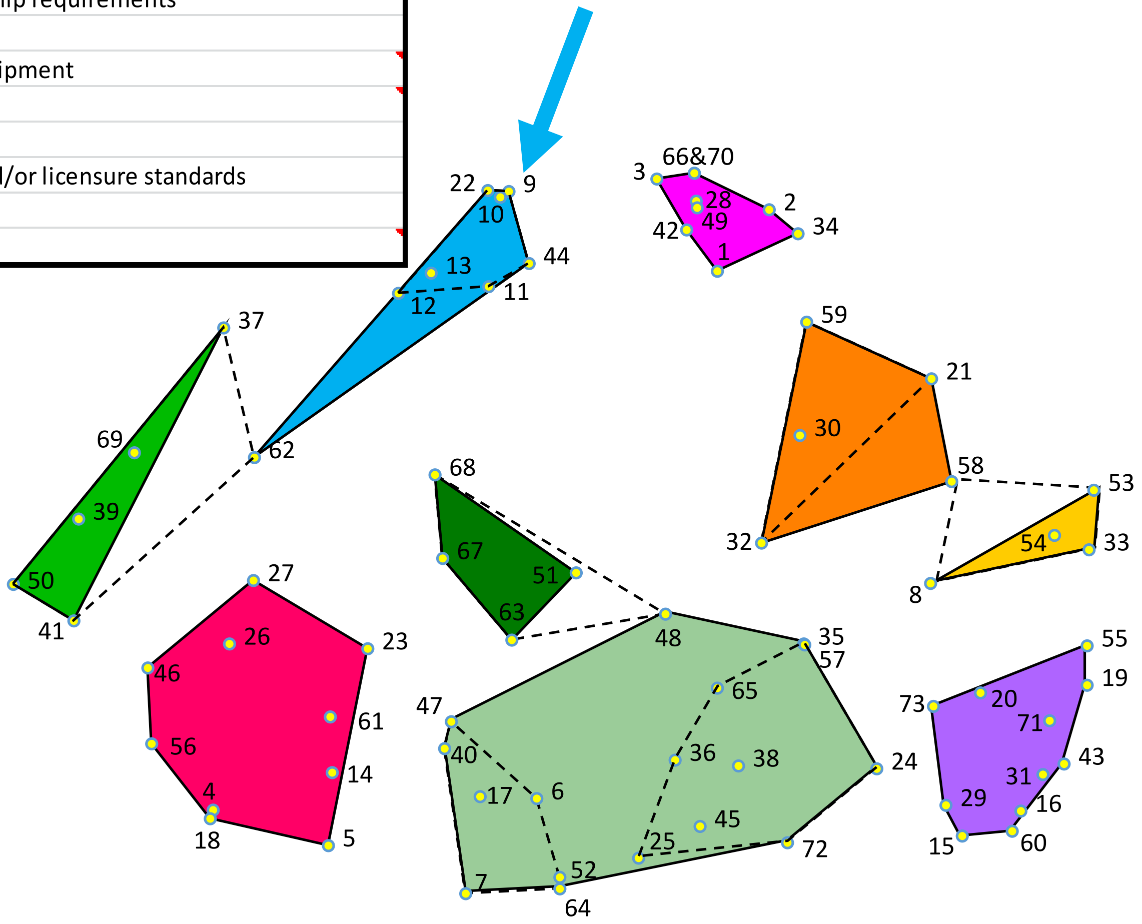

Go Zone Overview

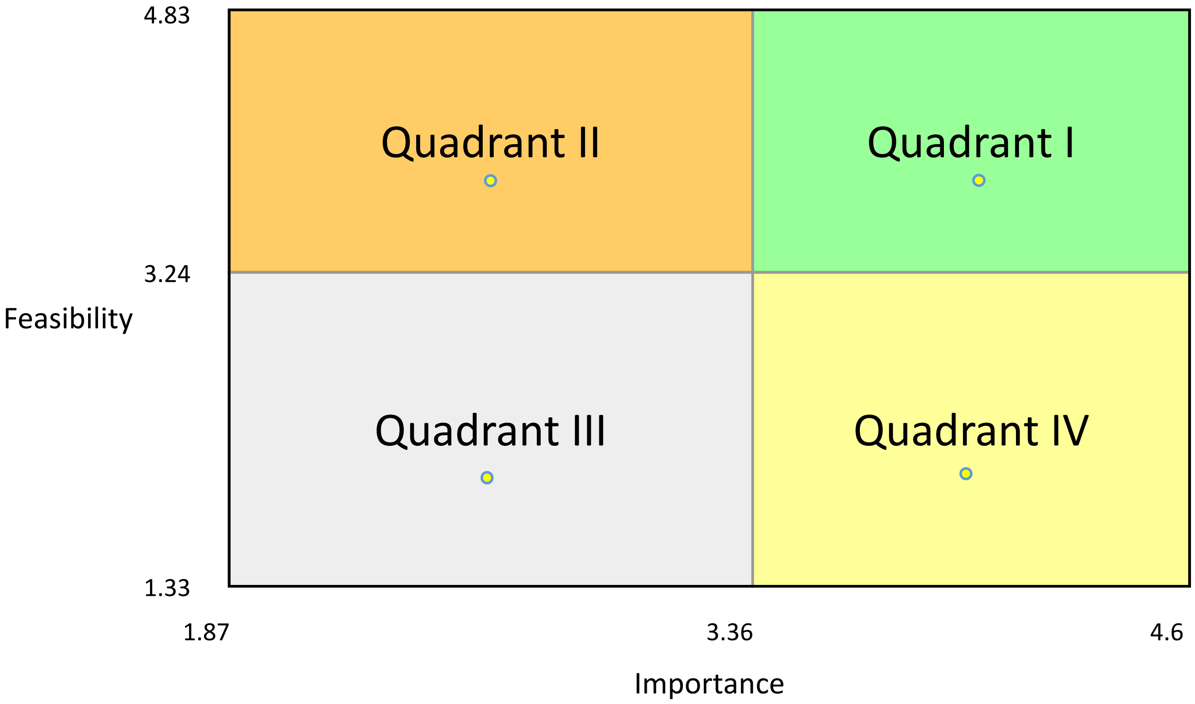

Rating Scale Anchors	
1	Relatively unimportant<br>Not at all feasible
2	Somewhat important<br>Somewhat feasible
3	Moderately important<br>Moderately feasible
4	Very important<br>Very feasible
5	Extremely important<br>Extremely feasible

Note. The range of the x and y axes reflect the range of mean values obtained for all 73 of the discrete implementation strategies for each of the rating scales. The plot is divided into quadrants on the basis of the overall mean values for each of the rating scales. Different means are calculated for each identifying the quadrants of each of the plots where only from the ratings of the strategies that compose the cluster under analysis (above the divisions reflect the means for all 73 strategies). Strategies falling in Quadrant I fall above the mean for both the importance and the feasibility ratings. Thus, these strategies are those where there was highest consensus regarding their relative high importance and feasibility. Conversely, Quadrant III reflects the strategies where there was consensus regarding their relative low importance and feasibility. Quadrants II and IV reflect strategies that were relatively high in feasibility or importance, respectively, but low on the other rating scale.

15. Conduct educational meetings
16. Conduct educational outreach visits
20. Create a learning collaborative
24. Develop academic partnerships
25. Develop an implementation glossary
40. Involve executive boards
58. Remind clinicians
60. Shadow other experts
65. Use an implementation advisor
71. Use train-the-trainer strategies
72. Visit other sites
73. Work with educational institutions

Go Zone-All

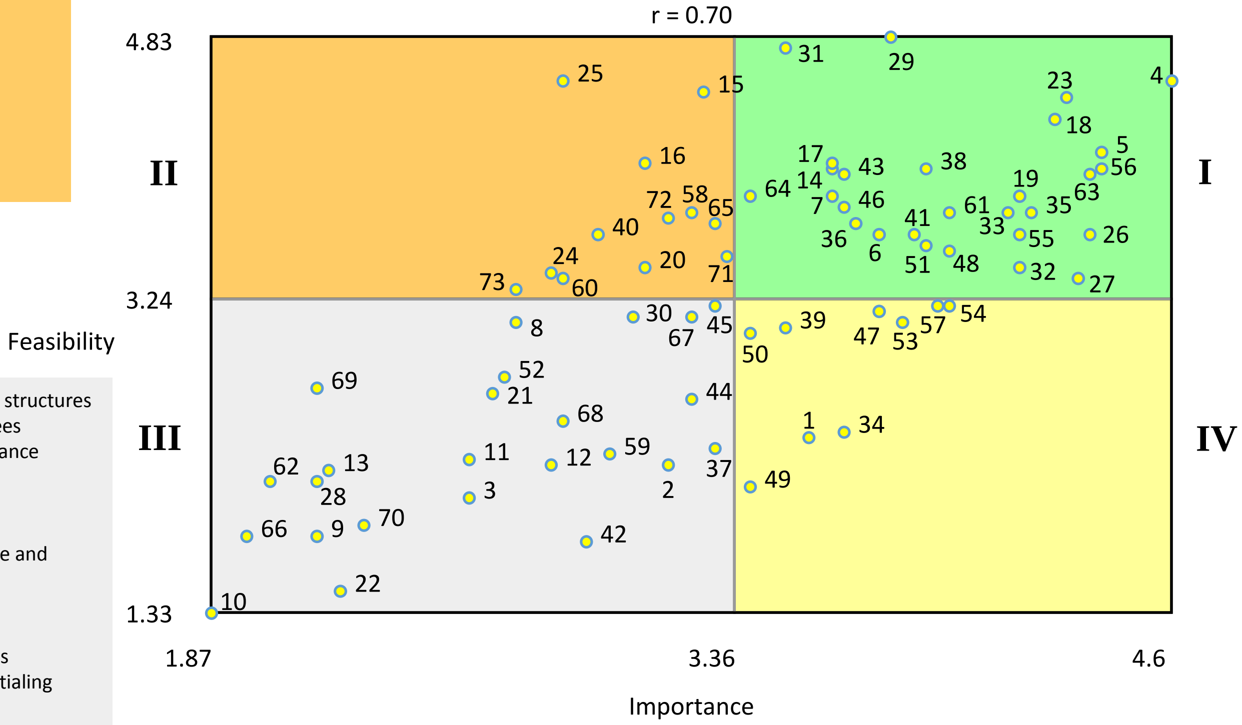

4. Assess for readiness and identify barriers and facilitators
5. Audit and provide feedback
6. Build a coalition
7. Capture and share local knowledge
14. Conduct cyclical small tests of change
17. Conduct local consensus discussions
18. Conduct local needs assessment
19. Conduct ongoing training
23. Develop a formal implementation blueprint
26. Develop and implement tools for quality monitoring
27. Develop and organize quality monitoring systems
29. Develop educational materials
31. Distribute educational materials
32. Facilitate relay of clinical data to providers
33. Facilitation
35. Identify and prepare champions
36. Identify early adopters
38. Inform local opinion leaders
41. Involve patients/consumers and family members
43. Make training dynamic
46. Obtain and use patients/consumers and family feedback
48. Organize clinician implementation team meetings
51. Promote adaptability
55. Provide ongoing consultation
56. Purposefully reexamine the implementation
61. Stage implementation scale up
63. Tailor strategies
64. Use advisory boards and workgroups

2. Alter incentive/allowance structures
3. Alter patient/consumer fees
8. Centralize technical assistance
9. Change accreditation or membership requirements
10. Change liability laws
11. Change physical structure and equipment
12. Change record systems
13. Change service sites
21. Create new clinical teams
22. Create or change credentialing and/or licensure standards
28. Develop disincentives
30. Develop resource sharing agreements
37. Increase demand
42. Make billing easier
44. Mandate change
45. Model and simulate change
52. Promote network weaving
59. Revise professional roles
62. Start a dissemination organization
66. Use capitated payments
67. Use data experts
68. Use data warehousing techniques
69. Use mass media
70. Use other payment schemes

1. Access new funding
34. Fund and contract for the clinical innovation
39. Intervene with patients/consumers to enhance uptake and adherence
47. Obtain formal commitments
49. Place innovation on fee for service lists/formularies
50. Prepare patients/consumers to be active participants
53. Provide clinical supervision
54. Provide local technical assistance
57. Recruit, designate, and train for leadership

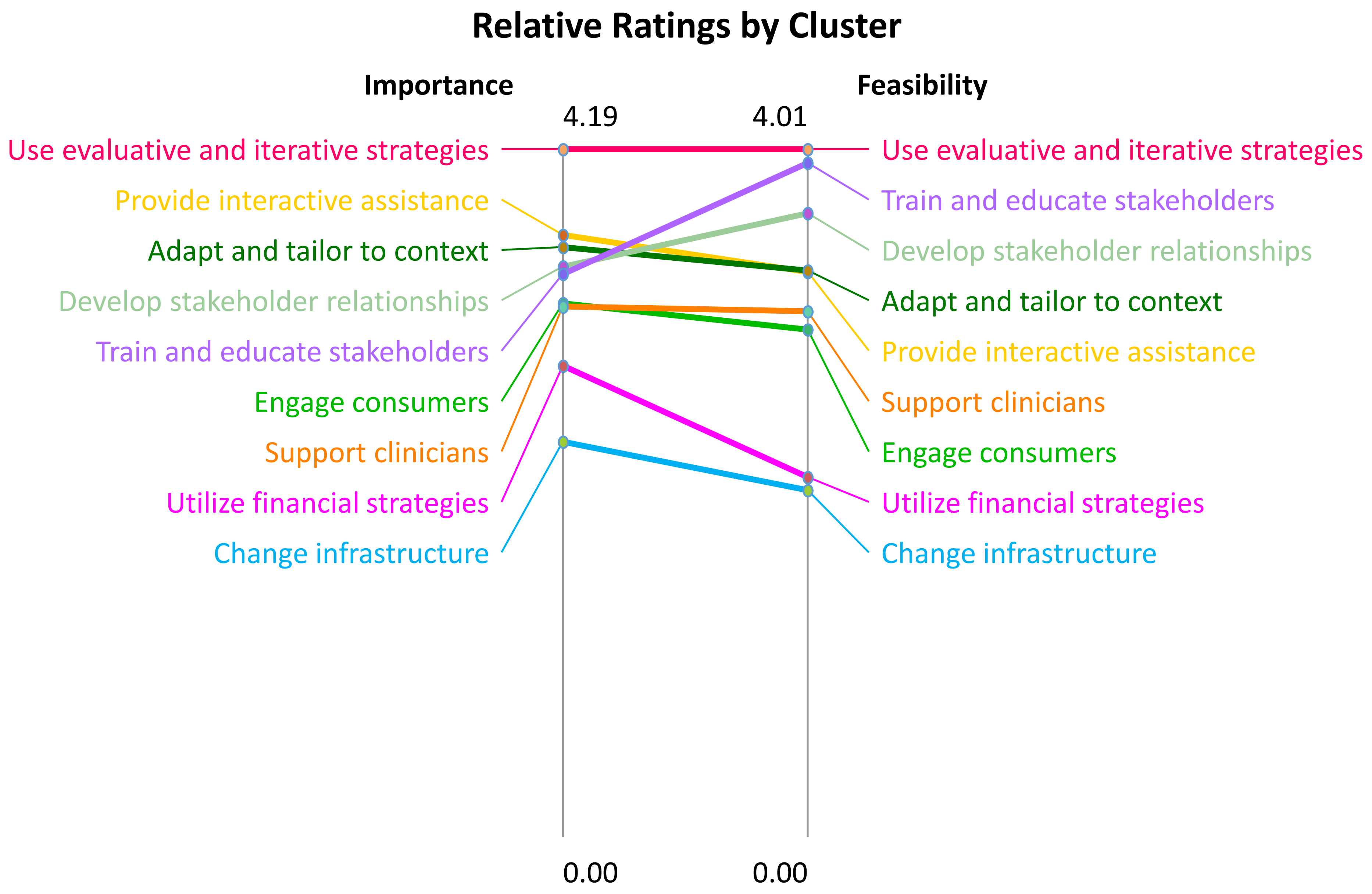

Relative Ratings by Cluster
Importance
Feasibility
4.19
4.01
Use evaluative and iterative strategies
Provide interactive assistance
Adapt and tailor to context
Develop stakeholder relationships
Train and educate stakeholders
Engage consumers
Support clinicians
Utilize financial strategies
Change infrastructure
Use evaluative and iterative strategies
Train and educate stakeholders
Develop stakeholder relationships
Adapt and tailor to context
Provide interactive assistance
Support clinicians
Engage consumers
Utilize financial strategies
Change infrastructure
0.00
0.00
